# Proceedings of Southern Dental Association

**Published:** 1871-09

**Authors:** O. J. Bond

**Affiliations:** Rec. Sect'y


### AMERICAN JOURNAL

OF

### DENTAL SCIENCE.

**Vol. V. THIRD SERIES--SEPTEMBER, 1871. No. 5.**

### ARTICLE I.

### *Proceedings of Southern Dental Association.*

The third annual meeting of this body was held in Charleston, S. C., April 12, 13, 14, and 15, 1871. The President, Prof. James S. Knapp, of New Orleans, in the chair. Session opened with prayer by Rev. Dr. Winkler. Welcome address by Dr. G. H. Winkler, and responded to by the President.

*Members present:* Drs. F. Y. Clark, E. A. Herman, John G. Angell, H. A. Lowrance, A. C. Ford, S. G. Holland, W. H. Burr, C. D. Alvigny, Wm. Reynolds, J. T. Campbell, E. Floyd, J. B. Patrick, and S. J. Cobb.

The following named persons were elected active members:

Drs. C. C. Patrick, G. H. Winkler, T. T. Moore, M. S. Kliuck, Wm. C. Wardlaw, B. A. Rodrigues, J. R Solomons, W. S. Brown, M. S. Hanckell, Henry C. Jones, H. M. Grant, J. F. Thompsen, A. M. Postley, T. F. Chupein, H. J. Royal, J. S. Jobson, Samuel A. White, B. C. Hart, C. B. Hutto, W. L. Reynolds, B. A. Muckenfuss, D. L. Boozer, G. F. S. Wright, T. W. Bouchier, O. J. Bond, A. B. Brookins, N. W. Kingsley, and W. R. Bull.

The following delegates were received, and also elected active members:

*American Dental Association*: Prof. W. H. Atkinson, New York.

*Pennsylvania Dental College:* Prof. Geo. T. Barker, Philadelphia.

WEDNESDAY AFTERNOON.

Prof. S. P. Cutler, Chairman of Committee on Histology and Microscopy, reported a paper on Microscopy of the teeth, which was read by Prof. Angell. By request Prof. Atkinson made some remarks on the same subject. He regretted the absence of Prof. Cutler, as he was thus deprived of the privilege of discussing some points in the report.

Dr. F. Y. Clark, Chairman of Committee on Dental Therapeutics, read a very interesting report, which was received and discussed.

Dr. E. Floyd, of N. C., said that he could not agree with Dr. Clark in regard to negroes having better teeth than the whites; he had never seen a negro twenty-five years old with a set of sound teeth; had seen white men forty and fifty years of age, with sound teeth.

Dr. Hanckell found a vast difference in the teeth of the negroes of the upper and lower country of South Carolina; those on the coast had the best teeth, the cause he supposed to be, that they do not separate the bran from the fine meal, as those of the upper country do.

Dr. Winkler said, that, in central Africa, where the negroes are large, healthy, and well developed, and live principally on the fruits of the chase, they have good teeth; he attributed it to their mode of living.

Prof. Barker regretted that he did not hear all the paper, as the portion that he did hear contained ideas similar to his own. He thought that we frequently arrived at erroneous conclusions by authorizing too much importance to food. It is necessary, not only that the food be good and nourishing, but that the organs have the power of making the proper appropriation. He mentioned two cases, which had lately come under his own observation; the one, a man eight feet one inch in height, had hard and well developed teeth, wearing like marble; the other a woman eight feet high, had small soft *leathery* teeth, without any hard osseous substance, but had a predominance of organic material. It was a subject that had enlisted his attention for several years, and he thought it well to advise the use of good nutritious food, and the observation of proper rules of health. He had never seen a set of perfect teeth. The nearest approach to perfection he had ever seen was in the mouth of a two-headed girl, with one body and four legs, who ate very little, but the appropriating organs were in good order.

Dr. J. B. Patrick said, the more strictly man lives in accordance with the laws of nature, the better his teeth will be. The tribe referred to by Dr. Winkler, have good teeth because they live on simple and nourishing diet; but when they come to this country, they degenerate.

WEDNESDAY EVENING.

Communications were read from Drs. Rambo, Morgan, McLain, and Redman, expressing regrets at not being able to to attend.

A communication was received from the South Carolina State Dental Association, inviting the members of the Southern Dental Association, to accompany them on an excursion up the Ashley River to the Phosphate works, and around Charleston Harbor.

The invitation was accepted.

The Discussion of Dr. Clark's paper was resumed.

Dr. Grant remarked that he had given the subject some thought, but not as much as it deserved. He thought that caries of the teeth could be inherited from either parent, and could be brought about by various causes, such as climate, clothing, and associations of the mind. That portion of the wheat which enters into the formation of bone, is, to a great extent, removed from flour as now manufactured. The poor, who subsist, principally, on corn bread and use but little flour, have better health and teeth than those whose principal diet consists of fine flour. The old slave who lived on coarse, cold diet, had better teeth than his master, and better than the house-servant who was fed from his master's table. He mentioned a negro girl, whose parents had good teeth, who had no inferior teeth: she had the superior teeth, but the processes seemed to be wasting away.

Dr. Cobb endorsed Dr. Clark's paper in many points. He believed that "like begets like," and that members of the same family could be identified by their dental organs, if the subject was thoroughly understood. He believed that disease was transmitted in consequence of the violation of some law of nature.

Dr. Ford thought that the case mentioned by Dr. Grant an abnormal one.

Dr. Moore wished to know how man had changed the shape of his dental organs.

Prof. Barker believed that we had modified *all* our organs by our degenerate manner of living, and thought that the two cases referred to by himself, illustrated that fact.

Dr. Reynolds did not think that the case without inferior teeth referred to by Dr. Grant, was hereditary. In regard to flour, he thought that much harm was done by giving too much of the bone producing substance, from an excessive use of which there is danger of an ossific deposit in the heart and arteries.

Prof. Atkinson. A full discussion of Dental Therapeutics can scarcely occupy much space, but if Dental Hygiene be allowable then the remarks already made have a decided and legitimate bearing. Intimate knowledge of the histology of the teeth is indispensable to enable us to understand the cause of imperfectly developed dental organs. There is but a single door of entrance to dental disease, properly so called, and that is *debility* of the formative elements denominated *cells*, which elaborate the enamel and dentine.

Imperfection of enamel and dentine may result from defficiency of the material out of which they are elaborated; but this is not properly called disease, for subsequently portions of both these tissues are well and fully formed as soon as the supply is brought in reach of the little builders of the dentinal and enamel tissue, the cells.

Deficiency of food, sunlight, and exercise are chargeable with all the imperfections in teeth that demand therapeutical knowledge and skill.

It is fortunate for the people, and for the profession of dentistry, that these tissues of the crowns of the teeth are so very lowly endowed with vital energy.

Were it otherwise, our efforts to remove debilitated and decayed portions, and to supply the place with a substitute, instead of arresting the process of decay, would only aggravate that condition, and render the salivation of the teeth an impossibility.

Dental therapeutics, then, consists in removing all sick dental tissue, be it enamel or dentine, and promptly supplying an indestructible substitute.

Dental hygiene consists in scrupulous cleanliness and abundant exercise of the organs of mastication.

Dr. Winkler thought that it was of great importance to the temporary teeth, in order to prevent contraction of the arch, and give ample room for the eruption of the permanent set.

Dr. Chupein regarded filling the deciduous teeth the best method of preserving them. He had evidence of this in the case of his own child. He filled the oral teeth when she was three years old; she is now eight, and the oral teeth are good, while the buccal are all gone.

Dr. Ford contended that cleaning teeth was not all that was necessary, that filling was essential. He filled a left lower molar of his child at two years of age; she is now eleven, and the tooth is still good.

Dr. Herman always treated children's teeth as simply as possible. If there is an abscess, and the tooth low, in a child of six or eight years of age, he would extract; in one of three or four, he would treat or extract, being governed by circumstances. He fills deciduous teeth with gold, and, if kept clean, they will last indefinitely.

Dr. Reynolds deprecated filling with amalgam.

Prof. Atkinson cleanses the cavity thoroughly and fills first with os. art., afterwards removing a portion of the oxychloride of zinc, and filling with amalgam. Treats abscess in children's mouths the same as in adults.

Dr. Cobb would fill with os-artificiel, amalgam, etc., as would best suit the case.

Dr. Clark cleanses the cavity as perfectly as possible and uses amalgam; in sensitive cases uses os-artificiel.

Dr. J. B. Patrick said that he was very cautious in treating alveolar abscess in children's mouths, (in order to preserve the teeth, thus preventing contraction of the arch), as it might be productive of serious injury. He mentioned a case in which had occurred, from the treatment of alveolar abscess, exfoliation of a portion of the maxilla, containing several teeth.

As the excursion would consume the whole of the following morning and afternoon, the Association adjourned till 8 P. M.

### THURSDAY EVENING.

The morning and afternoon having been consumed in the excursion, the Association assembled at 8 P. M., and was called to order by the President.

The Committee on Membership recommended Dr. S. H. Cartwright, M.R.C.S., of London, Eng., as an Honorary and Corresponding member of this Association, and on ballot he was elected.

The discussion of Dr. Clark's paper was resumed.

Prof. Atkinson. Almost any table in a civilized land affords a sufficient supply of the proper material to ensure a well developed denture. But the habits of nearly all civilized people are such as to prevent the full play of the formative functions of teeth and bones. The principal mistake is the over preparation of food by comminution and bad cooking. Indisposition to vigorous exercise, if indulged, will result in an inability to appropriate the earthy elements. This inability to appropriate the lime salts lays at the bottom of imperfectly developed enamel and dentine, to a much greater extent than the absence of these salts in our flour and other kinds of food.

There is but little really known in the direction of a clear apprehension and understanding of the histology and therapia of the tissues and organs of the human body. To get at a just conception of the part that hereditary influences play in health and disease, we must get rid of nearly all the doctrines of hereditary descent. Heretofore, they have been thought to be the product, in constitution of body and mind, of our immediate parents, that is, our father and mother. All this is fallacy. We are the product of many lines of descent, of many different races and bloods. Hence our teeth or other organs may partake of any of the various types of an illimitable ancestry.

No observing physician but has seen this exemplified extensively in every neighborhood. To such a degree does this hold good, that jealousies have arisen many times because of the close resemblance of features, of the teeth, and other organs, to uncle, cousin, or neighbor; when if the family history had been consulted, the origin of diversity and resemblance would have been vindicated in a legitimate way.

The longevity of tissues must be more closely studied before we are able to meet all indications in the therapia and hygiene of the teeth.

Dr. Cartwright thought that there were constitutional causes of decay, such as in *enciente* females. He said that it was generally conceded that decay could not take place in the dentine unless the enamel was affected; and that the free and proper use of the brush had done much towards preventing decay.

Dr. Winkler attributed decay to the action of acids. Food, permitted to remain around the teeth, ferments, softens the enamel, and offers a lodgement for vegetable fungi, and animal infusora, which are found in large quantities in favorable mouths. The color of the decay depends upon the rapidity of its progress.

The time having arrived for the selection of the next time and place of meeting, the last Tuesday in July, 1872, and Richmond Va., were unanimously chosen.

FRIDAY MORNING.

Dr. Kingsley, who had consented to demonstrate his method of supplying artificial palates, not being able to attend at the appointed time. Dr. Cartwright was requested to demonstrate the Anatomy and Physiology of the parts concerned, and the surgical operation resorted to for the purpose of relieving such inconvenience. Dr. C. though unexpectedly called upon, manifested, by his very clear illustrations, a perfect familiarity with the subject.

Dr. Kingsley having arrived, was called upon. He gave a minute, and very interesting description of his method of supplying artificial palates. The appliance consists of two pieces, made of hard and soft rubber; the latter was struck up with sectional dies made of type metal, and vulcanized; for one hour, at a temperature varying from 240° to 250°; one hour from 250° to 260°; and two hours from 260° to 270°.

Prof. Barker thought the appliance was more satisfactory when made in one piece, and all of hard rubber.

Dr. Cartwright said he had seen ten or twelve persons who were wearing artificial palates made by Dr. Kingsley, and they were suffering no inconvenience, except from the fact of the palates being a foreign body. Some of the parties could not be understood when talking without the artificial palates, but articulated distinctly with them.

Prof. Atkinson did not consider cases of cleft palate sufficiently numerous to induce every dentist to become an adept in the art of supplying the deficiency. He was glad to know where to send patients suffering from this deformity, as he did not like to undertake such cases.

Dr. J. B. Patrick said he had quite a number of cases of cleft palate from gun-shot wounds, and had always succeeded in restoring speech. He mentioned the case of a wounded officer, the shot passing from the anterior surface of the soft palate and uvula. A plate of gold was introduced, and he could speak distinctly, and smoke with comfort.

Dr. Clark cheerfully gave Dr. Kingsley the credit for his improvements in artificial palates, particularly those of soft rubber.

On motion of Dr. Winkler, a vote of thanks was tendered Dr. Kingsley for his cheerful compliance with the wishes of the Association in demonstrating his improvements in artificial palates.

On motion, the discussion of Dr. Clark's report was closed, and the regular order of business was resumed.

The other Committees not being ready to report, on motion Prof. Barker read a paper "On the Replanting of Teeth." *

Dr. Clark, referring to Prof. B.'s paper, objected to the injection of morphine as he considered it dangerous, a death having occurred in Savannah, Ga., from that cause.

Prof. Atkinson has heard of teeth being imported from Europe in brandy, and transplanted successfully. He considered the periosteum as simply a cementing tissue. Believed that the pulps may sometimes be united as demonstrated by Hunter, of England.

Dr. Moore had some experience in replanting teeth. Cited a case of an incisor tooth, which he could not cure; extracted it, clipped off the end, which was necrosed; filled the canal with gold and reinserted. There was no after trouble.

Dr. Moore mentioned the case of a molar tooth he had extracted, filled and reinserted with success. He knew that it was a common practice to extract and reinsert teeth which proved useful for years. Would like to know what length of time a tooth might remain out and be reinserted with success.

---

* Not left with the Rec. Secretary, but published in present No. of Journal.

Dr. Reynolds mentioned a case in which the Dens Sapientia had been removed, by mistake, and reinserted. Six years afterward, the lady consulted him in regard to paralysis of the eyelid, on the same side of the face as the replaced tooth. He examined the tooth but found nothing peculiar about it. The lady still suffered so much from her eye, that he extracted the tooth, and the eyelid recovered its integrity.

On motion of Dr. Cobb, the discussion was closed.

Dr. Herman read a paper on "The Importance of Elevating the Standard of the Dental Profession."

FRIDAY AFTERNOON.

On motion of Dr. Angell, a committee of three, consisting of Drs. Royal, Burr, and Lowrance, was appointed to draft suitable resolutions in regard to the death of Drs. Crow and Johnson. *

The subject of Dental Education was introduced.

Dr. Cobb thinks it is the most important of all subjects, and hopes every member will give it due consideration, as all of our notions and actions are based upon education.

We ought to keep up our Colleges, make them a success, and, in addition, endeavor to interest the people generally, in the profession. Our Dental Associations will do much towards elevating the profession by educating ourselves, as well as the public. He advocates a People's Dental Journal for circulation among the masses.

Dr. Winkler thought that our first object should be self-instruction, and then, that of the public. Dental Colleges are a great blessing; their principles are good, and they excite and stimulate the student to great effort. Dental Associations are also prolific of much good, but much of their success depends upon having active and energetic, working men on our committees.

Dr. Kingsley is radical on the subject. Many enter the profession who should be discouraged, as they are not suited to it, and would do better at something else.

---

* Published in present No. of Journal.

The discussion was then closed.

The subject of Operative Dental Surgery was discussed.

Prof. Atkinson referred to a difficult operation he had performed during clinics, in which he had used platina foil for filling the cavity. That metal suited this particular case better than gold, as it required less force to condense it, and adapted itself more readily to the walls of the cavity.

Dr. Clark, 1st Vice-President, being called to the chair, Prof. Knapp, by request, gave a very clear description of his method of manipulating with cylinders.

Dr. Herman formerly preferred cylinders for filling crown cavities, but now generally uses strips; can operate better with gold in this form when filling approximal cavities. He prefers gold to any other material, but sometimes uses amalgam. He never aims to make a display of his fillings, his only object is to save the tooth.

Dr. Clark manipulates gold in a variety of forms; adopts that one which he thinks best suited to each particular case.

FRIDAY EVENING.

The subject of Operative Dental Surgery was resumed.

Dr. Moore said that when a tooth was badly decayed, he did not hesitate to remove any frail portions of the enamel, as he considered the solid gold filling more desirable than frail enamel, though it might not look so well.

Prof. Angell.—Three things ought to be taken into consideration in filling a tooth, viz: usefulness, durability, and appearance; and that the last should be subservient to the other two. He always endeavored, when his judgement sanctioned it, to leave a tooth as near as possible to nature in shape, though he would not unnecessarily sacrifice any dentine or enamel, and preferred as little as possible of his fillings to be visible.

Dr. Reynolds mentioned the case of a lady, thirty-five years of age, who called upon him to operate upon a tumor situated in the anterior portion of the superior maxilla. All her teeth had apparently been extracted, as she stated, eight years previous, and was wearing a rubber plate. On examining the case, he found that the trouble proceeded from a cuspid tooth, embedded horizontally in the alveolar ridge. He removed the tooth and the *tumor* disappeared.

The Committee appointed to draft suitable Resolutions in reference to the death of Drs. T. J. Crow, and Warren Johnson, offered the following. (See Obituary Notices.)

An election for officers resulted as follows:

*President.*—Dr. F. Y. Clark, Savannah, Ga.

*First Vice-President.*—Dr. J. B. Patrick, Charleston, S.C.

*Second Vice-President.*—Dr. H. M. Grant, Abingdon, Va.

*Third Vice-President.*—Dr. E. Floyd, Fayetteville, N.C.

*Corresponding Secretary.*—Dr. A. C. Ford, Atlanta, Ga.

*Recording Secretary.*—Dr. O. J. Bond, Marion, S. C.

*Treasurer.*—Dr. W. G. Redman, Louisville, Ky.

The officers for the ensuing year were then duly installed.

On motion of Prof. Angell, Dr. S. Hape, of Atlanta, Ga., was elected an Honorary Member.

Prof. Angell being called upon for information in regard to establishing a Dental Journal, submitted a verbal report which was accepted.

New Orleans, La., was chosen as the place for the publication of the Journal, and Prof. S. P. Cutler, of New Orleans, La., elected editor.

The following named members were elected delegates to the American Dental Association:

G. H. Winkler, A. M. Postley, H. J. Royal, J. B. Patrick, H. A. Lowrance, W. H. Burr, Henry C. Jones, Jas. T. Thompson, H. M. Grant, W. R. Bull, W. S. Brown, Wm. C. Wardlaw, M. S. Hanckell, C. C. Patrick, D. L. Boozer, T. F. Chuypein, B. A. Rodrigues, J. P. H. Brown, G. F. S. Wright, B. A. Muckenfuss, and M. S. Jobson.

### SATURDAY MORNING.

A committee of three, consisting of Profs. Cutler, Knapp, and Angell, was appointed, with full power to take such steps as they may think proper, in carrying out the views of this Association in publishing a Dental Journal.

The following Committees were appointed for the ensuing year. (See August No. of Journal.)

Adjourned to meet in the city of Richmond, Va., the last Tuesday in July, 1872, at 10 o'clock, A. M.

O. J. Bond, Rec. Sect'y.

